# Multi-Objective Message Routing in Electric and Flying Vehicles Using a Genetics Algorithm

**DOI:** 10.3390/s23031100

**Published:** 2023-01-18

**Authors:** Muhammad Alolaiwy, Mohamed Zohdy

**Affiliations:** Electrical and Computer Engineering Department, Oakland University, Rochester, MI 48309, USA

**Keywords:** electric vehicles, UAVs, multi-objective optimization, genetics algorithm, EnFVs

## Abstract

With progressive technological advancements, the time for electric vehicles (EVs) and unmanned aerial vehicles (UAVs) has finally arrived for the masses. However, intelligent transportation systems need to develop appropriate protocols that enable swift predictive communication among these battery-powered devices. In this paper, we highlight the challenges in message routing in a unified paradigm of electric and flying vehicles (EnFVs). We innovate over the existing routing scheme by considering multi-objective EnFVs message routing using a novel modified genetics algorithm. The proposed scheme identifies all possible solutions, outlines the Pareto-front, and considers the optimal solution for the best route. Moreover, the reliability, data rate, and residual energy of vehicles are considered to achieve high communication gains. An exhaustive evaluation of the proposed and three existing schemes using a New York City real geographical trace shows that the proposed scheme outperforms existing solutions and achieves a 90%+ packet delivery ratio, longer connectivity time, shortest average hop distance, and efficient energy consumption.

## 1. Introduction

Applications of flying ad hoc networks (FANETs) and electric vehicular ad hoc networks (E-VANETs) have grown drastically in the past few years, spanning civil and military practices [1,2]. The intelligent transportation system (ITS) enables communication and connectivity in both these networks; however, the constraints and differences of these networks create challenges in developing an efficient message routing protocol. Unmanned aerial vehicles (UAVs) can move freely in all possible directions due to no geographical restrictions except obstructions. An EV has a relatively independent nature compared to a swarm of UAVs. Moreover, UAVs are energy-constrained but support higher data transmission capabilities, and EVs have to rely on either dedicated short-range communications (DSRC) or cellular networks. There exist several solutions which suggest utilizing FANETs to support E-VANETs’ communication, but no scheme considers both networks as a single paradigm [3,4]. The problem at hand is efficient message routing in a co-existent paradigm, where the ideal routing protocol should prioritize faster message transmission of UAVs without hindering their residual energy, while ensuring reliability and connectivity.

The authors in [4] propose a predictive message routing scheme for FANETs which identifies the current geographical coordinates of a target vehicle and deems its direction unchanged, analytically predicting the connectivity time. The scheme utilizes a quadratic equation-based time estimation but does not shed any light on the more common possibilities of no real solution or two real solutions. An interesting study in [5] uses Q-learning for a routing protocol that actively interacts with the environment and (re)adjusts its actions. A multi-objective routing scheme coined as EORB (energy-saver) in [6] considers node energy and buffer to choose the best route. The authors predict trajectory and velocity for communication and utilize a utility function for relay selection. On the other hand, ref. [3] introduced a UAV-assisted protocol for E-VANET, in which UAVs are responsible for a carry-and-forward mechanism for message delivery using Q-tables and rule-based fuzzy logic. An analytical connectivity time prediction-based routing scheme in [7] utilizes current geographical information and movement of the vehicles to choose a route that lasts longer. Routing in UAVs for specific applications such as public safety networks has been proposed in [8]. The authors use reinforcement learning to increase the lifespan of these aerial vehicles by considering energy as a routing metric. On the other hand, it is always possible to consider the original hop count-based routing scheme which not only ensures the minimum number of hops in a route but also results in a faster route selection [9]. Nevertheless, several existing solutions have tried utilizing SDN or a multi-objective routing for ground vehicles [10,11,12]. Moreover, routing in UAVs which considers metrics such as charging stations, VANET supporting networks, and trajectory-based systems has also been proposed [13,14,15,16,17,18,19]. An exhaustive review of existing schemes identified that a routing scheme should not consider a single objective while disregarding its byproduct’s impact. It should also be noted that none of the existing routing methods have considered the vehicular domain as a single entity.

**Figure 1:**
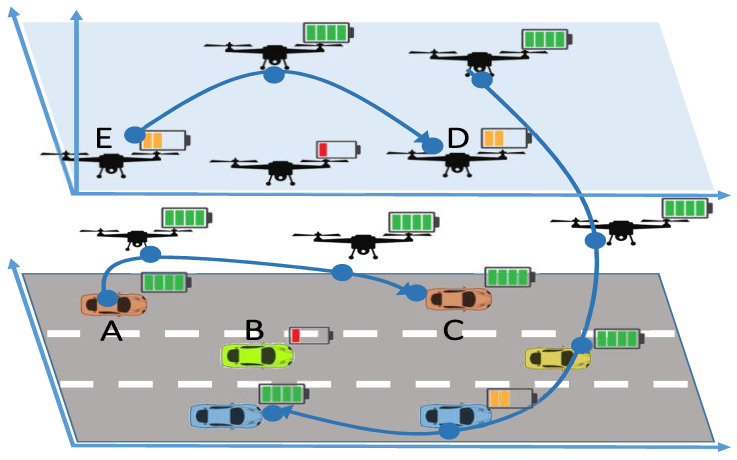
Proposed FA-VANET paradigm. **A**–**E** are dummy names for the vehicles.

A multi-objective scheme often requires evolutionary algorithms and techniques such as genetics algorithms (NSGA-I, II, etc.) which take input from a number of solutions and reduce it to the Pareto front (best possible solutions). Almost all the optimization methods consider a larger set of the population which increases the number of iterations, thus becoming space and time complex. In this paper, we propose a reduction in solution space before considering an optimization technique that identifies the Pareto front (set of optimal solutions in the objective function space) and chooses the best solution. The proposed genetics algorithm identifies the Pareto front and subsequently reduces or chooses the best possible solution. The proposed multi-objective routing method in a the proposed paradigm for mobile battery-powered vehicles employs two objective functions, data rate and residual energy. Considering data rate not only means higher data transfer but also confirms UAV’s inclusion in most communications routes, which leads to minimal blockage, better propagation, and faster communications. On the other hand, utilization of residual energy not only means sustaining the UAV’s power, but is also an ingenious way to avoid numerous routes employing the same communication devices. Moreover, the proposed scheme ensures reliability and identifies that the chosen path stays connected during the complete transaction. Figure 1 The novel optimal multi-objective solution has strong potential and applications in numerous similar problems. To the best of the authors’ knowledge, there exists no scheme which innovates with an analytical optimal solution for multi-objective routing in both high mobility networks of EnFVs. The following are the major contributions:

- An efficient and novel message routing scheme that considers battery-constrained mobile vehicles;
- Mathematical modeling of a multi-objective problem for message routing and a genetics algorithm;
- An efficient and reliable routing scheme for swift data transmission in a high mobility ad hoc environment;
- An exhaustive performance evaluation using real traces of simulation for urban mobility (SUMO) and OpenStreetMap (OSM); and
- 90%+ successful packet delivery as compared to existing solutions.

The organization of the remainder of the paper is as follows. Section 2 analytically models the proposed unified E-VANET and FANET environment system. In Section 3, we discuss the proposed algorithms and our solution. The proposed scheme and existing state-of-the-art routing solutions have been comparatively evaluated in Section 4. Section 5 concludes the paper.

## 2. System Model

Our system model includes vehicles (UAV or EV) with important properties such as 3D location, represented by $l_{i}\left(x_{i},y_{i},z_{i}\right)$ along with velocity of $\gamma_{i}$. These mobile vehicles can communication with each other within a transmission range $R_{i}$. The straight line distance $\Delta D_{i,j}$ between a vehicle *i* and *j* can be identified using $\Delta D_{i,j}=\sqrt{{\left(x_{j}-x_{i}\right)}^{2}+{\left(y_{j}-y_{i}\right)}^{2}+{\left(z_{j}-z_{i}\right)}^{2}}$. It is assumed that all EVs are at z=0 position in the 3rd dimension, whereas UAVs can have a positive *z* value.

With the assumption of uniform velocity and unchanged direction for all vehicles at time t = 0, the future position of a vehicle can be predicted as ($\Delta D_{i,j}\left(t_{0}+\tau \right)$) at time $t_{0}+\tau$ using the current position at the current time and movement vector, as given below:

(1) $$\begin{array}{c} \Delta D_{i,j}\left(t_{0}+\tau \right)=\sqrt{{\left[\left(x_{j}+v_{x,j}\times \tau \right)-\left(x_{i}+v_{x,i}\times \tau \right)\right]}^{2}}+\sqrt{{\left[\left(y_{j}+v_{y,j}\times \tau \right)-\left(y_{i}+v_{y,i}\times \tau \right)\right]}^{2}}+\\ \sqrt{{\left[\left(z_{j}+v_{z,j}\times \tau \right)-\left(z_{j}+v_{z,j}\times \tau \right)\right]}^{2}} \end{array}$$

A movement vector shows the acceleration of a vehicle in the direction of any one, two, or all of the three dimensions, in a unit of time (ex. seconds). The UAVs functionality allows the availability of the movement vector ($v_{x,i},v_{y,i},v_{z,i}$) in their beacon or hello messages; however, in EV, the only relatable available information is the velocity ($\gamma_{i}$) and acceleration direction ($\theta_{i}$). EVs share periodic basic safety messages (BSM) under the DSRC communication protocol, which contains location, velocity, and direction information. The movement vector for an EV can be extracted by considering the top view where the north always points to $90{}^{\circ }$, and by dividing the total $360{}^{\circ }$ into quadrants of equal size (Qs). Here, $Qs=45{}^{\circ }$ is considered, which creates eight quadrants and an offset of Qs/2=22.5. Thus, if a vehicle’s direction angle is between $0{}^{\circ }∼22.5{}^{\circ }$ or $337.5^{\circ }∼360{}^{\circ }$, then a positive value with a velocity of $\gamma_{i}$ on the x-axis is considered for $v_{x,i}$. A simple movement direction metric $\theta_{i}^{\prime }=\left(\theta_{i}+\mathrm{Offset}\right)/Qs$ is formulated, which identifies the movement of EV in a positive or negative value over the x-axis, y-axis, or both axis. The movement of an EV on the x-axis and y-axis in a single time unit with an unchanged direction can be estimated as:

(2) $$v_{x,i}=\left\{\begin{array}{cc} \left(+1\right)\times \gamma_{i} & ,\;\mathrm{if}\;0\leq \theta_{i}^{\prime }<2\;or\;\theta_{i}^{\prime }>7\\ \left(-1\right)\times \gamma_{i} & ,\;\mathrm{if}\;3\leq \theta_{i}^{\prime }<6\\ \left(0\right)\times \gamma_{i} & ,\;\mathrm{if}\;2\leq \theta_{i}^{\prime }<3\;or\;6\leq \theta_{i}^{\prime }<7 \end{array}\right\}$$

(3) $$v_{y,i}=\left\{\begin{array}{cc} \left(+1\right)\times \gamma_{i} & ,\;\mathrm{if}\;1\leq \theta_{i}^{\prime }<4\\ \left(-1\right)\times \gamma_{i} & ,\;\mathrm{if}\;\theta_{i}^{\prime }\geq 5\\ \left(0\right)\times \gamma_{i} & ,\;\mathrm{if}\;0\leq \theta_{i}^{\prime }<1\;or\;4\leq \theta_{i}^{\prime }<5 \end{array}\right\}$$

with the $v_{z,i}=0$, velocity ($\gamma_{i}$) and vehicle direction ($\theta_{i}$), a movement vector ($v_{x,i},v_{y,i},v_{z,i}$) can be formulated for an EV (*i*) to employ in Equation (1), using Equations (2) and (3). Practically, it is unlikely that a vehicle keeps on moving in the same direction, but even a short time using the proposed prediction is enough to successfully communicate with reliability.

Reliable data transfer is identified by our coined metric of $\omega_{i,j}$, which considers data rate and the size of the data $Dt_{i,j}$ between two vehicles, as $\omega_{i,j}=\frac{\tau_{i,j}\times Tx_{i,j}}{Dt_{i,j}}$, where $\tau_{i,j}$ is the connectivity time between vehicle *i* and *j*.

The value of $\omega_{i,j}$ represents transmission between vehicles *i* and *j*, such that $\omega_{i,j}<1$ depicts unreliable and incomplete data transmission. Algorithm 1 outlines a novel module to estimate the reliability ($\omega_{i,j}$) of a link between vehicle *i* and *j* by incrementally exploring connectivity time $\tau_{i,j}$. The process terminates execution when it achieves reliable connectivity ($\omega_{i,j}\geq 1$) or both vehicles move out of communication range ($R_{i,j}$). A quadratic solution can also predict $\Delta D_{i,j}\left(t_{0}+\tau \right)$, but through extensive trial and error evaluation, it has been identified that often there exist multiple or no solutions, which lead to unreliable prediction. Moreover, Algorithm 1 considers mobility metrics to estimate connectivity time while avoiding the need for any probe message to gather network metrics (throughput, bandwidth, etc.). The proposed reliability check only considers the data in hand (BSM messages, etc.), and does not require any network information for prediction. The route-reliability procedure in Algorithm 1 increases connectivity time by α (0.5, 1, etc.) time units (seconds) with every iteration and continuously checks that both vehicles are within communication range ($\Delta D_{i,j}\left(t_{0}+\tau \right)<R_{i,j}$). The algorithm also converges if the reliability metric ($\omega_{i,j}$) reaches 1, which translates to a successful transmission of data ($Dt_{i,j}$). Through this ingenious process, the reliability of a communication link can be easily identified without the additional overhead of probe messages.
**Algorithm 1** The algorithm implemented in each communicating vehicle 1:Initiate and distribute a vehicle discovery message 2:For all responses regarding the target vehicle 3:**if** no direct link exists between source and the destination **then** 4:    **for all** loop-free responses with target vehicle **do** 5:        Route $\omega_{s,d}=min\left(\omega_{s,1},\omega_{1,2},\ldots ,\omega_{l,d}\right)$, 6:        **while** calculate all $\omega_{i,j}$ for hops between s and d **do** 7:           **while** $\Delta D_{i,j}\left(t_{0}+\tau \right)<R_{i,j}\;\mathrm{and}\;\omega_{i,j}<1$ **do** 8:               $\tau_{i,j}=\tau_{i,j}+\alpha$ 9:               Find $\Delta D_{i,j}\left(t_{0}+\tau \right)$ for hop i and j.10:               Identify $\omega_{i,j}=\frac{\tau_{i,j}\times Tx_{i,j}}{Dt_{i,j}}$11:           **end while**12:        **end while**13:        **if** $\omega_{s,d}<1$ **then** Remove the response as unreliable14:        **end if**15:    **end for**16:    Call multi-objective genetics algorithm () with remaining responses messages17:**else**18:    Direct link between source and the destination supersedes all possible paths.19:**end if**20:Return the optimal route as transmission path.

Moreover, the average data rate $\left(O^{1}\right)$ of the source, and all intermediate nodes in a route (*p*) between the source (*s*) and destination (*d*) can be calculated as:

(4) $$O_{s,d}^{1}=\left(sum(Tx_{s,i},Tx_{i,j},\ldots ,Tx_{l,d})\right)/hops$$

On the other hand, the minimum residual energy $\left(O^{2}\right)$ of any vehicle in a route (*p*) between the source (*s*) and destination (*d*) can be calculated as:

(5) $$O_{s,d}^{2}=min\left(\xi_{s,i},\xi_{i,j},\ldots ,\xi_{l,d}\right),$$

where $\xi_{i,j}$ is residual energy of vehicle *i*. A reliable route ($\omega_{i,j}\geq 1$) having the highest $O^{1}$ and $O^{2}$ values, prioritizes UAVs in the path whilst keeping their residual energy afloat.

## 3. Multi-Objective Vehicular Routing in EnFVs

Let G(V,E) be a directed graph of a communication paradigm containing UAVs and EVs, represented as set V with E, communication edges. In a multi-hop environment, a communication route exists if it meets the criteria of specified constraints, C. If there exist multiple routes between a source and a destination, then a chosen route from problem space must satisfy the following objective functions $\left(O^{1},O^{2}\right)$:

(6) $$\begin{array}{c} argmax_{p\in M(s,d)}O^{1}\\ argmax_{p\in M(s,d)}O^{2}\\ s.t.\;C1:\;\forall \;argmin_{p}\left(\omega_{s,1},\omega_{1,2},\ldots ,\omega_{m,d}\right)>1\\ C2:\;0<\forall \xi_{i}\leq 100\\ C3:\;\forall Tx_{i}>0, \end{array}$$

where M(s,d) is the solution space of possible paths between *s* and *d*, considering all preliminary inquisitions. The objective functions $O^{1}$ and $O^{2}$ are defined in Equations (4) and (5), respectively. Algorithm 1 outlines that a sender vehicle discovers and evaluates multiple routes towards a destination. A broadcast shares route request (RReq) messages to all connected vehicles. Each route reply message from the destination or any intermediate vehicle having a route to the destination includes additional information for the proposed scheme, as follows: (1) $min\omega_{i,d}=min\left(\omega_{i,j},min\omega_{j,d}\right)$, (2) $min\xi_{i,d}=min\left(\xi_{i,j},min\xi_{j,d}\right)$, (3) $sumTx_{i,d}=sum\left(Tx_{i,j},sumTx_{j,d}\right)$, (4) hop count.

Each route reply message includes additional information which helps in route evaluation. A route reply message includes $min\omega_{i,d}=min\left(\omega_{i,j},min\omega_{j,d}\right)$, $min\xi_{i,d}=min\left(\xi_{i,j},min\xi_{j,d}\right)$, $sumTx_{i,d}=sum\left(Tx_{i,j},sumTx_{j,d}\right)$ and hop count.

After receiving all route reply messages, if there is any direct route between source and destination, then the source vehicle selects the route and terminates further route evaluation. On the other hand, the algorithm calculates $\omega_{s,d}$ for each route and discards all unreliable paths ($\omega_{s,d}<1$). Subsequently, the remaining routes are passed to a multi-objective optimal route algorithm which calculates both objective functions ($O^{1},\;O^{2}$) to represent each route. In the end, the source device chooses the best route based on objective functions from available routes. Algorithm 2 describes that using population M(s,d) in Equation (6); a multi-objective problem under defined constraints can be formulated wherein all objective functions values are normalized between [0, 1]. The multi-objective problem formulation in Equation (6) supports any number of objectives and constraints. The proposed scheme considers two objective functions for the maximum average data rate, $O^{1}$ and the highest minimum residual energy, $O^{2}$ with three constraints. Along with reliability ($\omega_{s,d}<1$), $O^{1}$ ensures that a route with UAVs has priorities, whereas $O^{2}$ considers avoiding UAVs with low residual energy. Considering that both objectives have a contradictory nature, a multi-objective solution allows a fair trade-off wherein both objectives have a higher value. Normally, a multi-objective optimization algorithm calculates the Pareto front for all solutions, which is a set of non-dominant solutions such that no other solution exists that outperforms. However, all the solutions on the Pareto front are not favorable to all objectives. Moreover, genetic algorithms and traditional evolutionary algorithms identify and reduce the Pareto front for a solution set. The proposed scheme considers reliability and complete message delivery using ω factor and reduces the solution space before the genetics algorithm. It is worth noting that the population (*M*(*s*,*d*)) in the proposed Algorithm 2 is already filtered out using the reliability check of Algorithm 1 and requires only minimal processing, thus becoming the lightweight multi-objective optimal procedure. Algorithm 2 outlines the complete process of the proposed multi-objective optimization using a genetics algorithm. First, the reduced population (M(s,d)) is each assigned objective functions with constraints defined in Equation (6). The solution space mutates with crossover and selection to transform into an updated population. The iterative process converges when a solution (or Pareto front) has been identified.
**Algorithm 2** Multi-objective genetics algorithm 1:M(s,d) is the solution space population having all possible but filtered routes 2:$O^{1}$ and $O^{2}$ are maximized along with constraints C 3:Problem modeling according to Equation (6) 4:**if** constraint satisfied with optimal solution **then** 5:    Output the solution 6:**else** 7:    Perform selection, mutation and crossover 8:    Update population 9:    Repeat Step 210:**end if**

## 4. Performance Evaluation

**Figure 2:**
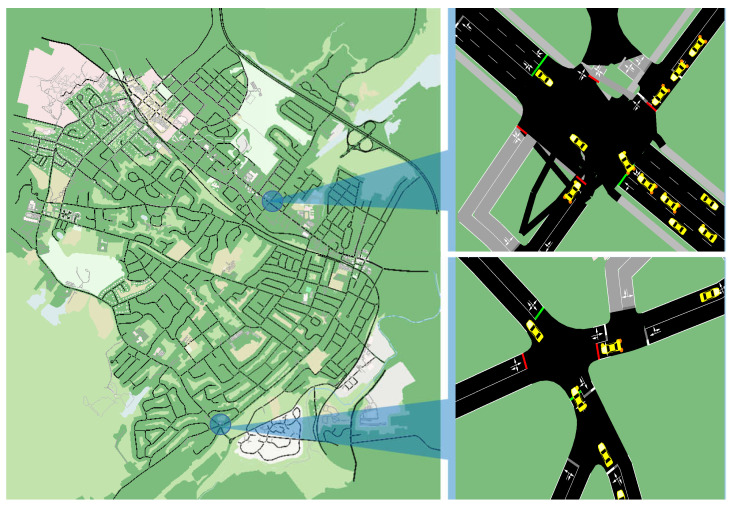
SUMO simulation trace (New York City).

The proposed GA-based multi-objective solution is evaluated exhaustively in comparison with three existing state-of-the-art routing schemes. We believe that the energy consumption of these battery-powered mobile vehicles is a critical issue and should be considered in communication technologies. However, it is important that the numerical evaluation of the systems should be realistic (nearly) and depict the environment well. Our exhaustive comparative evaluation considers real traffic traces of New York City (4 × 3 km^2^) housing 400∼900 EV and 1000 UAVs. The geographical environment for the simulation is illustrated in Figure 2. Each of the EVs and UAVs is expected to be equipped with a battery and two communication modules, along with their complete functionality kits. Each vehicle is assumed to have two connectivity interfaces to transmit data (40 Mb). One communication interface for EVs has a communication range of 250 m with a 20 Mbps data rate, and another for UAVs has a 500 m range and a 30 Mbps data rate. The EV mobility pattern follows SUMO simulator commands [7], whereas the UAVs adhere to a random waypoint with a speed of 10∼50 m/s [20]. A comprehensive custom-built event-driven python simulation is designed, which populates SUMO trace, containing EV with UAVs while generating 100 s of packets, each for a random source and destination. The simulation ran on a Dell PowerEdge T430 with Intel Xeon processor E5-2600 v3 product family 12-Core, with a clock speed of 3.5 GHz. The simulator accumulates all possible paths from a source to the destination. The proposed scheme and three existing solutions chose one route out of the accumulated paths, depending on their routing parameters and conditions. Finally, the simulator calculates performance metrics and repeats the process for more than 100 packets during each timestamp with a different number of total vehicles.

**Figure 3:**
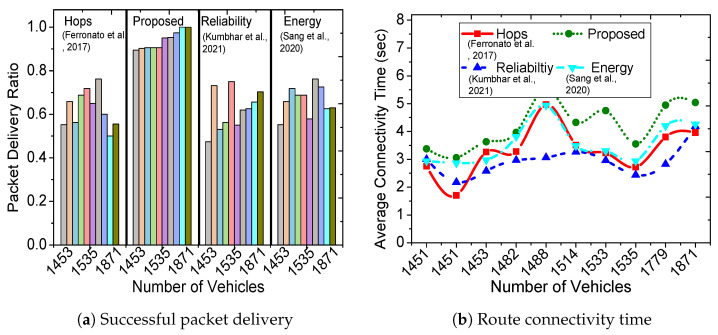
Comparative evaluation of the proposed with hops [9], reliability [7] and energy [6] routing.

**Figure 4:**
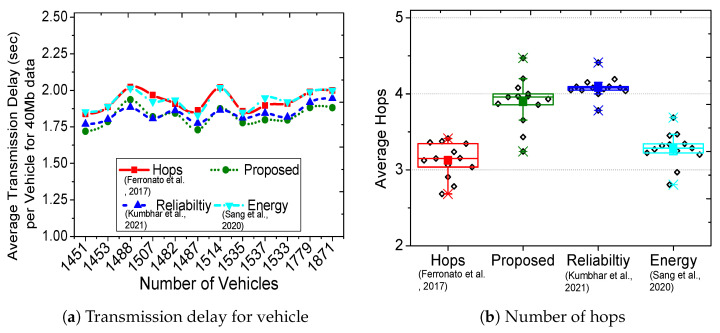
Comparative evaluation of the proposed with hops [9], reliability [7] and energy [6] routing.

**Figure 5:**
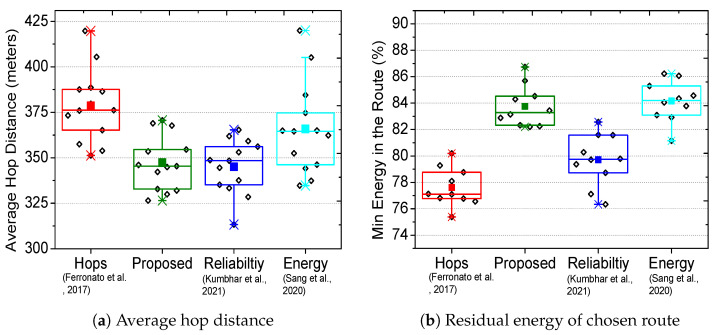
Comparative evaluation of the proposed with hops [9], reliability [7] and energy [6] routing.

Figure 3a shows the packet delivery ratio for the proposed scheme and three existing solutions (hop-based [9], reliable routing [7], and energy-saver [6]). The comparative analysis of the proposed scheme in Figure 3a considers hop-based only as a legacy benchmark; the actual comparison is between the latest connectivity time-based routing (reliable routing [7]) and multi-objective based routing schemes (energy-saver [6]). Each section of bars represents the evaluation of timestamps containing 1453∼1871 vehicles, where the proposed scheme achieves more than 90% success. Interestingly, with the increase in the number of vehicles, the hop-based performance degrades to 50% success, whereas reliable routing achieves better results (70% packet delivery). On the other hand, the energy-saver successfully delivers the packets for 50∼70% of the time. Figure 3b demonstrates that the chosen route in the proposed scheme remains connected for a longer period of 3∼6 s, whereas hop-based remained connected for 1∼5, energy-saver for 3∼5 and reliable routing for 1∼4 s. A longer connectivity time results in a higher packet delivery ratio and reduces the possibility of incomplete transmissions. The exhaustive experiments use real traces of New York City that include intersections, turns, and various road structures; however, the proposed scheme still outperforms existing solutions and achieves high packet delivery with longer connectivity. In Figure 4a, the average transmission delay per vehicle in the chosen route is shown, where almost all schemes show a delay between 1.7∼2 s. This delay depends on the transmission capability of the vehicles, and it does not include propagation and queuing delay. Nevertheless, our scheme outperforms existing solutions and achieves the lowest transmission delay per vehicle. However, the transmission delay per vehicle is a result of an extra hop in the proposed scheme’s chosen route, as illustrated in Figure 4b. The chosen routes in the proposed scheme and reliable routing have at most four hops, whereas hop-based and energy-saver have ∼3 hop count. Figure 5a shows that the average distance between each hop in the proposed scheme and reliable routing is the lowest, which directly translates to better communication, lower propagation delay, and minimal transmission losses. The proposed scheme and energy-saver consider residual energy as a major route selection metric, thus resulting in as much as 87% for the minimum energy in chosen route. The metric in Figure 5b shows the residual energy of a vehicle in the route with the lowest battery. The hop-based and reliable routing chose routes regardless of energy consideration, thus achieving a minimum energy for the intermediate vehicles of as low as 75%.

The proposed scheme outperforms three state-of-the-art routing methods on various fronts and achieves better packet delivery, longer connectivity, shorter transmission delay, shortest hop distance, and energy conservation. The EVs periodically share required information using BSM, whereas the UAVs need to include movement vectors with the route reply messages. The modified route reply messages require additional information delivery for at least four float values. Moreover, the proposed scheme requires analytical processing of the routing metrics ($O^{1},O^{2},maxHV$, and κ). However, the lightweight calculation is trivial, and requires lesser efforts than or almost similar efforts to most existing schemes. The proposed multi-objective optimal routing reduces solution space for evolutionary algorithms and Pareto front calculations while achieving exceptional results. The evaluations suggest that the trade-off of additional processing for higher gains and reliable communication is reasonably acceptable.

## 5. Conclusions

In this paper, a novel and unified multi-objective optimal routing scheme for FANETs and E-VANETs (EnFVs) is proposed, which considers reliability, data rate, and residual energy as routing metrics. The proposed solution reduced the solution space for optimization algorithms and formulated an analytical optimal solution that offers faster optimization. Exhaustive simulation using a New York City traffic trace demonstrated that the proposed scheme achieved a 90% packet delivery ratio with longer route connectivity (3∼5 s) and shorter hop distance, thus outperforming three existing state-of-the-art solutions.

## Data Availability

Not applicable.

## Abbreviations

The following abbreviations are used in this manuscript:

UAVs	Unmanned aerial vehicles
EV	Electric vehicles
FANETs	Flying ad hoc networks
VANETs	Vehicular ad hoc networks
SUMO	Simulation for urban mobility
OSM	OpenStreetMap
EnFVs	electric and flying vehicles
